# C/EBPβ-LAP*/LAP Expression Is Mediated by RSK/eIF4B-Dependent Signalling and Boosted by Increased Protein Stability in Models of Monocytic Differentiation

**DOI:** 10.1371/journal.pone.0144338

**Published:** 2015-12-08

**Authors:** René Huber, Thomas Panterodt, Bastian Welz, Martin Christmann, Judith Friesenhagen, Andreas Westphal, Daniel Pietsch, Korbinian Brand

**Affiliations:** Institute of Clinical Chemistry, Hannover Medical School, 30625 Hannover, Germany; University of Cologne, GERMANY

## Abstract

The transcription factor C/EBPβ plays a key role in monocytic differentiation and inflammation. Its small isoform LIP is associated with proliferation at early premonocytic developmental stages and regulated *via* mTOR-dependent signalling. During later stages of (pre)monocytic differentiation there is a considerable increase in the large C/EBPβ isoforms LAP*/LAP which inhibit proliferation thus supporting terminal differentiation. Here, we showed in different models of monocytic differentiation that this dramatic increase in the LAP*/LAP protein and LAP/LIP ratio was accompanied by an only modest/retarded mRNA increase suggesting an important role for (post)translational mechanisms. We found that LAP*/LAP formation was induced *via* MEK/RSK-dependent cascades, whereas mTOR/S6K1 were not involved. Remarkably, LAP*/LAP expression was dependent on phosphorylated eIF4B, an acceleratory protein of RNA helicase eIF4A. PKR inhibition reduced the expression of eIF4B and C/EBPβ in an eIF2α-independent manner. Furthermore, under our conditions a marked stabilisation of LAP*/LAP protein occurred, accompanied by reduced chymotrypsin-like proteasome/calpain activities and increased calpastatin levels. Our study elucidates new signalling pathways inducing LAP*/LAP expression and indicates new alternative PKR functions in monocytes. The switch from mTOR- to RSK-mediated signalling to orchestrate eIF4B-dependent LAP*/LAP translation, accompanied by increased protein stability but only small mRNA changes, may be a prototypical example for the regulation of protein expression during selected processes of differentiation/proliferation.

## Introduction

The transcription factor CCAAT/enhancer binding protein β (C/EBPβ) plays an important role in the regulation of proliferation and differentiation as well as inflammatory and metabolic processes and cancer [1–3]. The expression of the intronless C/EBPβ gene is regulated on several levels, i.e. *de novo* mRNA synthesis, alternative translation, posttranslational modification, nuclear import/export, and protein/protein interactions [2–4]. Alternative translation—starting at three different in-frame start codons of the C/EBPβ mRNA—leads to the expression of three different isoforms. The complete protein and a slightly shortened C/EBPβ variant are designated as liver-enriched activating proteins (LAP* and LAP) which provide transactivation capacity and are associated with differentiation, whereas liver-enriched inhibitory protein (LIP) represents a strongly shortened isoform which is transcriptionally inactive and supports proliferation [5]. This procedure of protein formation is regulated by several elements, e.g. by weak Kozak consensus sequences around the initiation codons for C/EBPβ-LAP* and -LAP and an optimal Kozak context for LIP [5] in combination with the leaky scanning mechanism of the ribosome [6]. In addition, translation of a short ex-frame upstream open reading frame (uORF; located between the first and the second regular start codon) impedes the formation of LAP, but is essential for LIP expression [5].

Moreover, alternative translation of C/EBPβ isoforms is controlled by different signalling modules regulating the activity of several translation factors. The translation of LIP is mainly regulated by mammalian target of rapamycin (mTOR) which directly increases the amount of accessible eukaryotic translation initiation factor (eIF) 4E [5] and by association of CUG binding protein 1 (CUG-BP1) with the C/EBPβ mRNA [7]. On the other hand, synthesis of the larger C/EBPβ isoforms can be increased under certain cellular conditions by protein kinase R (PKR) *via* phosphorylation of eIF2α [5]. Several mRNA binding proteins, e.g. CUG-BP1 which binds within the uORF [7] or calreticulin which interacts with GC-rich stem loop structures upstream of the uORF initiation codon are involved in the regulation of C/EBPβ mRNA translation [8]. Further mRNA binding proteins may also influence the C/EBPβ protein amount *via* indirect mechanisms. For instance, binding of HuR to the C/EBPβ mRNA may result in an enhancement of mRNA stability [9] potentially enabling a prolonged phase of translation.

Several reports suggest that the expression of C/EBPβ is also regulated at a posttranslational level by proteolysis [10]. For example, it has been reported that the lifespan of C/EBPβ proteins is restricted by proteasome- or calpain-dependent proteolytic mechanisms [11, 12]. During the response to endoplasmatic reticulum stress, the LAP/LIP ratio appears to be mainly regulated *via* a decrease or increase in both protein synthesis and stability predominantly of LIP [11]. Moreover, it has been proposed that directed proteolytic cleavage of LAP* and LAP contributes to the generation of LIP [13]. The *in vivo* significance of this phenomenon, however, is not clear yet, since protein isolation may be accompanied by partial proteolytic degradation of full-length C/EBPβ *in vitro* [14].

Previous experiments from our laboratory demonstrate that activation of the fms-like tyrosine kinase 3 (FLT3) receptor by stimulation with its ligand FL or the presence of the internal tandem duplication (ITD) mutation leads to a marked expression of proproliferative LIP and a decrease in the LAP/LIP ratio [15]. In this context, physiological receptor activation induces LIP expression *via* mTOR-dependent signalling pathways, whereas dysregulated ITD-induced LIP formation is mediated by constitutive 90 kDa ribosomal protein S6 kinase (RSK-)dependent signalling [15]. It has also been shown that stimulation of the FLT3-receptor is associated with progenitor cell proliferation at early stages of myeloic and premonocytic differentiation [16]. In contrast, the ITD mutation plays a role in the pathogenesis of leukemia [16]. During later stages of (pre)monocytic differentiation, we detected a dramatic increase in the large C/EBPβ isoforms LAP* and LAP which inhibit proliferation thereby supporting final differentiation [17]. The mechanisms which are responsible for this remarkable LAP*/LAP-driven shift in the LAP/LIP ratio during differentiation are not known. Here we demonstrate that this increase in LAP*/LAP proteins is accompanied by only slightly induced mRNA levels which suggests an important role for (post)translational mechanisms. The increase in LAP*/LAP is mediated by MEK/RSK- but not mTOR-dependent signalling which orchestrates eIF4B-dependent translation, a process also suggesting alternative PKR functions. In addition, under differentiation-supporting conditions we find a significantly increased C/EBPβ protein stability and reduced proteasome as well as calpain activities. These data may represent a prototypical example for the coordinated regulation of protein expression under certain conditions of differentiation and cell homeostasis.

## Materials and Methods

### Isolation and differentiation of primary human monocytes

Blood samples from healthy donors were provided by the Institute of Transfusion Medicine (Hannover Medical School). Informed donor consent was obtained and the experiments were approved by the Hannover Medical School ethics committee in accordance with the Declaration of Helsinki. Human monocytes were isolated by Biocoll (Biochrom AG, Berlin, Germany) density gradient centrifugation using LeucoSeptubes (Greiner Bio-One, Frickenhausen, Germany). For negative cell selection, biotin-conjugated monoclonal antibodies and magnetic antibiotin microbeads (Monocyte Isolation Kit II; Miltenyi Biotec, Bergisch Gladbach, Germany) were applied according to manufacturer’s instructions. Purified cells were cultured in 24-well Costar ultra-low attachment plates (Sigma Aldrich, St. Louis, USA) at a density of 1x10^6^ cells (final volume: 1 ml) in RPMI 1640 (Biochrom) which in the standard protocol was supplemented with 7.5% FCS (Lonza, Basel, Switzerland), 100 U/ml penicillin, 100 m g/ml streptomycin (Biochrom), 2% OPI media supplement (Sigma Aldrich), 1% Life Technologies MEM nonessential amino acids solution (Invitrogen, Darmstadt, Germany, and 100 ng/ml recombinant human (rh) macrophage colony stimulating factor (M-CSF; BioLegend, San Diego, USA). Following an adherence step for 1 h, cells were washed three times with supplemented medium and cultured at 37°C with 5% CO_2_. In each case, endotoxin contamination of the reagents was excluded using the Limulus Amoebocyte Lysate assay (Lonza) (˂10 pg endotoxin/ml).

### Flow cytometry

Purity of isolated monocytes (>90%) was assessed by dual cell labeling (30 min, 4°C, dark) using Alexa Fluor405-CD45 (Invitrogen), APC-CD14, and PE-CD16 (BD Biosciences, Heidelberg, Germany) antibodies. Contaminations with other cell types were excluded using APC-CD3, PE-CD19, FITC-CD56 (BD Biosciences), and FITC-CD15 (Invitrogen) antibodies. Detection was performed using a FACSCanto II flow cytometer and FACSDiVa software (BD Biosciences).

### Cell lines

THP-1, MonoMac 6 (MM-6), HeLa (DSMZ, Braunschweig, Germany), and immortalised murine macrophage-like cells [18] were cultured in RPMI 1640 medium containing 7.5% FCS, 100 U/ml penicillin, and 100 μg/ml streptomycin at 37°C with 5% CO_2_ [19]. The MM-6 medium was also supplemented with 1% OPI Media Supplement. THP-1 cells were seeded at a density of 1x10^6^ / 1.5 ml (12-well plates; NUNC) or 2x10^6^ / 3 ml (6-well plates; Sarstedt, Nümbrecht, Germany). MM-6, HeLa, and macrophage-like cells were alternatively seeded at 5x10^5^ / 2.5 ml in 6-well plates.

### Isolation and differentiation of murine bone marrow-derived cells

C57BL/6J mice were housed under specific-pathogen-free conditions at the central animal facility of Hannover Medical School. Research involving animals, including animal housing and breeding, was carried out according to institutional guidelines approved by the Lower Saxony State Office for Consumer Protection and Food Safety (“Niedersächsisches Landesamt für Verbraucherschutz und Lebensmittelsicherheit”). Protocols were compliant with §4 of the German Animal Protection Law (“Tierschutzgesetz”). Since experiments were performed *ex vivo* on isolated bone marrow cells, no further permits were required.

Primary bone marrow-derived cells were isolated on ice with sterile PBS from the rear leg bones of sacrificed mice. Erythrocytes were eliminated using prewarmed sterile erythrocyte lysis buffer (RT, pH 7.3) containing 155 mM NH_4_Cl, 16 mM Na_2_CO_3_ (Merck Millipore, Darmstadt, Germany), and 1 mM EDTA (Applichem, Darmstadt, Germany). Following centrifugation (350 g, 5 min, 4°C) and removal of the supernatant, 6x10^6^ primary cells were cultured in petri dishes containing 10 ml medium (RPMI 1640 including 10% FCS, 50 U/ml penicillin, 50 μg/ml streptomycin, 1% nonessential amino acids (Invitrogen), 2% L-glutamine, and 1% sodium pyruvate (Sigma Aldrich)) at 37°C with 5% CO_2_. Differentiation towards macrophages was induced by treatment with 20 ng/ml recombinant murine (rm) M-CSF (Biolegend) up to 7 d.

### Reagents

Differentiation of THP-1 and MM-6 cells was performed using Phorbol 12-myristate 13-acetate (PMA; Merck Millipore) or 1-α, 25-dihydroxyvitamin D_3_ (VitD3; Sigma Aldrich). For the inhibition of kinases and proteases, the following inhibitors were applied: S6K1 inhibitor DG2 (S6K-I), PKR inhibitor (PKR-I; 6,8-Dihydro-8-1H-imidazol-5-ylmethylene)-7H-pyrrolo[2,3-g]benzothiazol-7-one), ALLN (N-[N-(N-Acetyl-L-leucyl)-L-leucyl]-L-norleucine), MG-132, Calpain-Inhibitor V (Merck Millipore), rapamycin (mTOR-I; Sigma Aldrich), UO126 (MEK-I; Cell Signaling, Danvers, USA), BI-D1870 (RSK-I; Axon Medchem BV, Groningen, Netherland), and Cathepsin-Inhibitor I (Applichem). For Western Blotting, specific antibodies directed against RSK, phosphorylated (p-)RSK (T573), ERK1/2, p-ERK1/2 (T202/Y204), rpS6, p-rpS6 (S335/236), eIF4B, p-eIF4B (S422), eIF4A, eIF2α, p-eIF2α (S51), PKR (Cell Signaling), p-PKR (T446; Abcam, Cambridge, UK), V5 Tag (Invitrogen), C/EBPβ, Calpastatin, Histon H4 (Santa Cruz), Actin, glyceraldehyde-3-phosphate dehydrogenase (GAPDH), or TATA box-binding protein (TBP; Sigma Aldrich) were used in combination with secondary horseradish peroxidase (HRP-)coupled antibodies (Vector Laboratories, Peterborough, UK). All media and reagents were of the best available grade and routinely tested for endotoxins with the Limulus Amoebocyte Lysate assay (Lonza).

### Protein extraction, SDS-PAGE, and Western blot analysis

For SDS-PAGE and Western blot analysis, whole cell extracts were used. Cells were lysed in extraction buffer (150 mM NaCl (Merck Millipore, Darmstadt, Germany), 25 mM MgCl_2_ (Roth, Karlsruhe, Germany), 50 mM Tris-HCl (Applichem, Darmstadt, Germany), 10% Glycerol (Merck Millipore), 1% NP-40, and 3 tablets protease-inhibitor (Roche, Rotkreuz, Switzerland) per 50 ml) and additionally sonicated to also obtain nuclear proteins (10 s, 20% power; Bandelin, Berlin, Germany). Protein concentrations were determined *via* Bradford Assay (BioRad, Hercules, USA). SDS-PAGE was performed using 12% Tris/Glycin-SDS-polyacrylamide gels (Biostep, Jahnsdorf, Germany). Subsequently, proteins were transferred onto 45 μm nitrocellulose membranes (BioRad) using the Western blot technique (transfer buffer: 200 mM glycin (Applichem) and 25 mM Tris-base, pH 8.3). For washing of Western blot membranes, Tris buffered saline (TBS) containing 0.1% Tween (Sigma-Aldrich) (TBST) or phosphate buffered saline (PBS) with 0.1% Tween (PBST) were used. After blocking with 5% skim milk (Merck Millipore) or bovine serum albumin (BSA, Roche) in TBST or PBST, membranes were incubated with the specific primary antibodies at 4°C overnight. Following incubation with HRP-coupled secondary antibodies, protein bands were visualised using HRP substrates enhanced chemoluminescence (ECL) or SuperSignal West Femto (Thermo Fisher, Bonn, Germany) and densitometric analyses were performed using the Bio-imaging system MF-ChemiBis 3.2 and the TotalLab TL100 analysis software (Nonlinear Dynamics, Newcastle, UK) [15, 17, 20].

### Calculation of LAP/LIP ratios

Intensities of LAP*, LAP, and LIP protein bands were quantified by densitometry. Subsequently, the LAP/LIP ratio was calculated by dividing the combined LAP* and LAP value (denoted as LAP in the LAP/LIP ratio) by the respective LIP value [15].

### RNA extraction, cDNA synthesis, and qRT-PCR

Extraction of total RNA was performed with the RNeasy Mini Kit (Qiagen, Hilden, Germany) according to the manufacturer’s instructions. RNA concentrations were determined using a Nanodrop ND-1000 (Peqlab, Erlangen, Germany). cDNA synthesis and qRT-PCR were performed as previously described [15, 17, 20]. For mRNA expression analysis, two specific primer pairs were used for C/EBPβ cDNA detection (5’ mRNA section at pos. 192–300, spanning parts of the 5’-UTR and the beginning of the coding region: 5’- GTTCATGCAACGCCTGGTG -3’, 5’- CAGTCCGCCTCGTAGTAGAA -3’; central mRNA section at pos. 975–1145, located in the coding region: 5’- AGCAAGGCCAAGAAGACCGTGGA -3’, 5’- ACCTTCTTCTGCAGCCGCTCGT -3’) and expression was normalised to the mRNA expression levels of housekeeping gene GAPDH (5’- AGGTCGGAGTCAACGGAT -3’, 5’- TCCTGGAAGATGGTGATG -3’). The qRT-PCR was performed using a LightCycler 480 (Roche).

### Determination of mRNA stability

To determine the stability of the endogenous C/EBPβ mRNA, THP-1 cells were treated with 1 μg/ml Actinomycin D (Life Technologies) in the absence or presence of 100 nM PMA for 0, 0.5, 1, 2, 4, 6, and 24 h. Subsequently, the C/EBPβ mRNA amount was assessed using q-RT-PCR.

### Vector generation

To generate a tagged C/EBPβ-expressing plasmid, the 1837 bp full length C/EBPβ sequence (i.e. including 5’- and 3’-UTR; located at chromosome 20; GenBank^TM^ accession number: NC_000020.11) was used. Initially, a fragment containing the 5-’UTR and the C/EBPβ coding sequence was cloned into the pcDNA^TM^3.1/ V5-His B (+) vector backbone (Invitrogen) in front of the Tags *via* the *Eco*RI and *Xho*I restriction sites using the respective restriction enzymes (NEB, Ipswich, USA) and T4 DNA ligase (Invitrogen). The 3’-UTR was subsequently cloned behind the Tags and the internal vector stop codon using the *Pme*I site. Finally, the C/EBPβ stop codon was modified by Quick Change Site-Directed Mutagenesis Kit (Agilent, Santa Clara, USA) to enable the translation of the whole protein including the Tags (V5-Tag, His-Tag) using the primers: 5’- CTGCTCGCCTCCTCCGGCCACTGCTACTCGAGTCTAGAGGGCCCGCGG-TTCGAA -3’ and 5’- TTCGAACCGCGGGCCCTCTAGACTCGAGTAGCAGTGGCCGGA-GGAGGCGAGCAG -3’.

### Transfection experiments

Transfection of THP-1 and HeLa cells was performed using XtremeGene HP (THP-1) or XtremeGene 9 (HeLa) transfection reagent (Roche) according to the manufacturer’s instructions. In short, freshly grown THP-1 or HeLa cells were seeded at 2x10^5^ cells in 500 μl RPMI 1640, 10% FCS in 12-well (THP-1) or 6-well (HeLa) plates. XtremeGene reagent was preincubated with 25 nM RSK- or eIF4B-siRNA (Santa Cruz, Dallas, USA) or a combination of 100 nM C/EBPβ-V5 expression plasmid and 25 nM eIF4B-siRNA (ratio transfection reagent to nucleic acids: 3:1) for 30 min in RPMI 1640. The cells were then incubated with 100 μl of the transfection mixture per well for 24 h. Subsequently, cells were lysed and the respective protein amounts were assessed using SDS-PAGE/Western blot [20].

### Determination of protein stability

For examining the C/EBPβ protein stability in differentiating monocytic cells, THP-1 or MM-6 cells were treated with PMA for 24 h or with VitD3 for 72h and primary human monocytes were differentiated for 3 d. Following pretreatment of cells with Cycloheximid (CHX; Merck Millipore) for 30 min, LAP*/LAP protein levels were assessed at the indicated time points. To assess the influence of RSK on protein stability, PMA-treated THP-1 cells (24 h) were incubated with CHX ± RSK-I (pretreatment phase: 0.5 h) and LAP*/LAP were analysed at the presented time points. To assess the influence of protease inhibitors on C/EBPβ protein stability, THP-1 cells were treated with CHX ± ALLN, MG-132, Calpain-Inhibitor V, or Cathepsin-Inhibitor I up to 16 h. Afterwards, LAP*/LAP protein amounts were assessed at the indicated time points using SDS-PAGE/Western blot and protein half-life was determined by densitometry [17].

### Protease Assays

For determination of proteasomal and calpain activities, the Proteasome-Glo Assay System (Promega) was used. Optimal time points and amounts of whole cell extracts applied for analysis were determined in preliminary time course and dose response experiments. THP-1 cells were treated ± 100 nM PMA or a combination of PMA and RSK-I up to 24 h and primary human monocytes were cultivated for 72 h. Whole cell extracts were isolated at the indicated time points. The extracts were incubated with Proteasome-Glo^TM^ reagents for the assessment of chymotrypsin-, trypsin-, and caspase-like proteasomal activities as well as calpain activity for 30 min in 96 well plates (Corning Inc., Corning, USA). The emitted light was subsequently measured using the OrionL Microplate Luminometer.

## Results

### Marked increase in the larger C/EBPβ isoforms LAP*/LAP and LAP/LIP ratio in different models of monocytic differentiation

**Fig 1:**
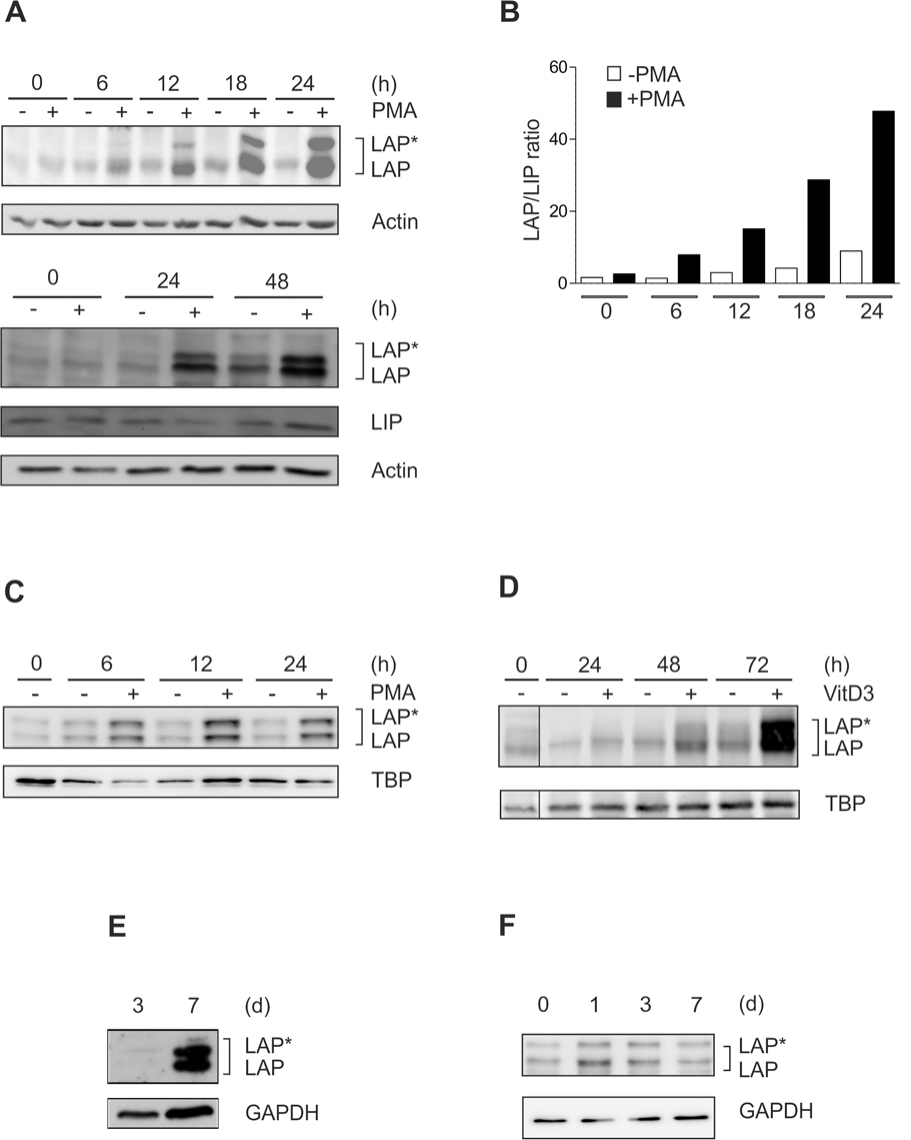
Dramatic increase in C/EBPβ-LAP*/LAP proteins and LAP/LIP ratio in different models of monocytic differentiation. (A) THP-1 monocytic cells were incubated with 100 nM PMA up to 48 h, and the levels of the larger C/EBPβ isoforms (LAP*/LAP) and the small isoform LIP were monitored by Western blot analysis using whole cell extracts (loading control: actin; n = 6). (B) LAP*/LAP and LIP levels of a representative experiment were analysed by densitometry. The LAP/LIP ratio was calculated by adding LAP* and LAP and dividing this value by LIP. (C) Monocytic MM-6 cells were incubated with 100 nM PMA up to 24 h, and the levels of LAP*/LAP were measured (loading control: TBP; n = 3). (D) THP-1 cells were incubated with 100 nM VitD3 up to 72 h and the levels of LAP*/LAP were determined (loading control: TBP; n = 6). Please note that a control lane has been removed (indicated by a thin line). (E) Murine bone marrow-derived cells were differentiated to macrophages using 20 ng/ml rmM-CSF up to 7 d and LAP*/LAP were measured at day 3 and day 7 (loading control GAPDH; n = 3). (F) Primary human monocytes were differentiated up to 7 d, and LAP*/LAP levels were assessed at day 0, 1, 3, and 7 (loading control: GAPDH; n = 5).

As a model for monocytic differentiation, THP-1 monocytic cells were incubated with PMA up to 48 h [19], and the levels of the C/EBPβ isoforms LAP*, LAP, and LIP were monitored. These experiments revealed a dramatic increase in the larger C/EBPβ isoforms LAP*/LAP and the LAP/LIP ratio during this time interval (Fig 1A and 1B). A similar LAP*/LAP elevation was observed in a second monocytic model, i.e. MM-6 cells (Fig 1C). Following incubation with VitD3, which is an alternative stimulus for the induction of monocytic differentiation, we also found a marked increase in C/EBPβ-LAP*/LAP proteins albeit at a later time point (Fig 1D). A prominent increase in the larger C/EBPβ isoforms was also detected in murine bone marrow-derived cells driven towards monocytic differentiation by incubation with rmM-CSF up to 7 d (Fig 1E). Consistent with the marked increase in C/EBPβ-LAP*/LAP levels in differentiating premonocytic cells shown above, significant levels of LAP*/LAP protein were detected in isolated primary human monocytes, albeit with an only minor further increase during ongoing differentiation up to 7 d (Fig 1F). Therefore, to study the regulation of the dramatic increase of the larger C/EBPβ isoforms, we focused in the subsequent experiments on premonocytic models of monocyte differentiation.

### Modest and retarded increase in C/EBPβ mRNA

**Fig 2:**
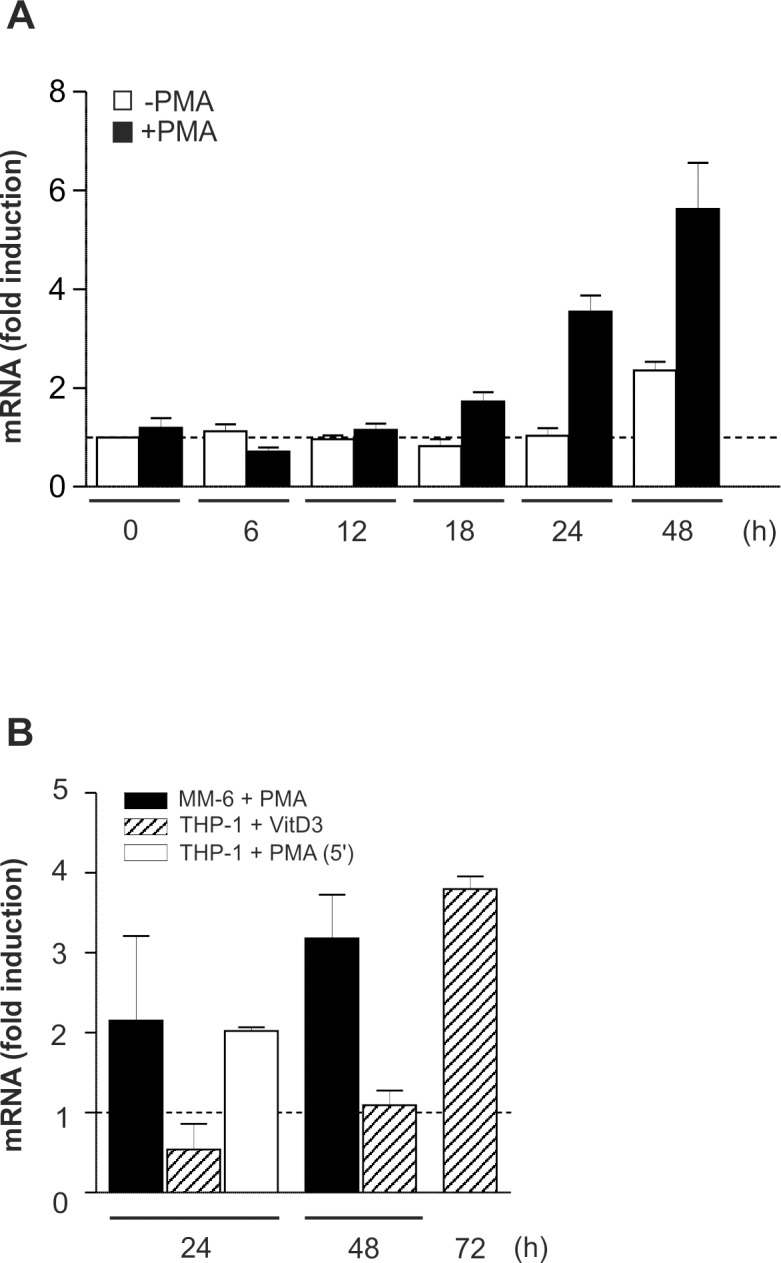
Modest and retarded increase in C/EBPβ mRNA under differentiation-inducing conditions. (A) Under conditions as shown in Fig 1A, the expression of C/EBPβ mRNA in THP-1 cells was monitored by qPCR using a C/EBPβ-specific primer pair in the central mRNA section, and GAPDH was used as a housekeeping gene control (mean±SD; n≥6, measured in duplicates). The level of C/EBPβ mRNA in unstimulated cells at 0 h was defined as one (dashed line). (B) Under conditions as described in Fig 1A, 1C and 1D, C/EBPβ mRNA expression was measured in monocytic cell lines and analysed as described in A. THP-1 + VitD3, MM-6 + PMA: mean±SD; n = 9 each (duplicates). THP-1 + PMA (5’): qRT-PCR using an additional C/EBPβ-specific primer pair in the 5’ section of the mRNA; mean±SD; n = 4 (duplicates).

Next, the expression of C/EBPβ mRNA was examined in THP-1 and MM-6 cells under differentiation-inducing conditions. These experiments revealed an only modest, somewhat retarded elevation of mRNA levels in the presence of PMA or VitD3, respectively (Fig 2A and 2B), and a comparable mRNA stability in non-differentiated *versus* treated cells (data not shown) which suggested a major role for (post)translational regulatory mechanisms. To exclude position-dependent effects potentially tampering the correct determination of C/EBPβ mRNA expression, e.g. by the occurrence of different C/EBPβ mRNA species based on alternative transcription start site usage, an additional C/EBPβ-specific primer pair was used located in the 5’ section of the mRNA (Fig 2B).

### C/EBPβ-LAP*/LAP expression is mediated by MEK/RSK-dependent signalling

**Fig 3:**
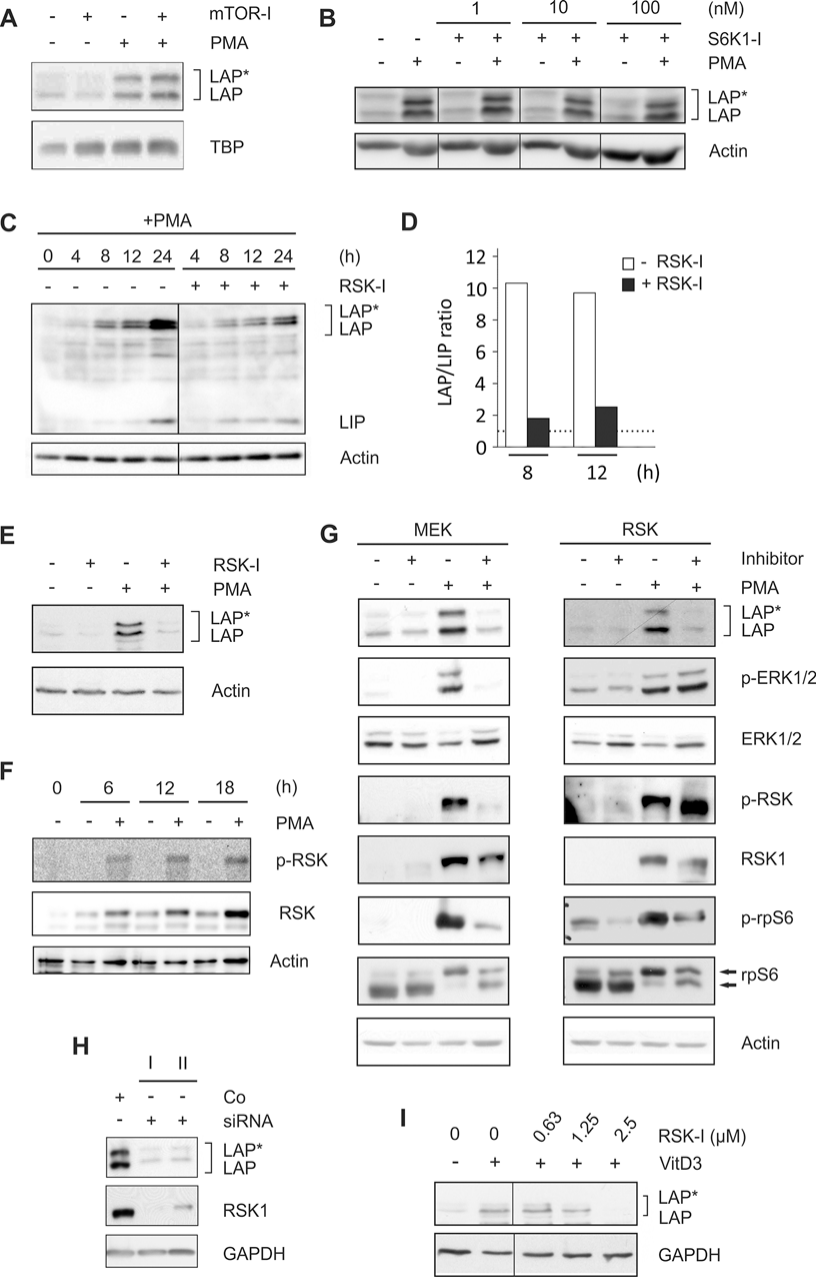
Expression of LAP*/LAP is regulated by MEK/RSK-dependent signalling pathways. (A) THP-1 monocytic cells were coincubated with the mTOR inhibitor (mTOR-I) rapamycin (100 nM) and/or 100 nM PMA for 24 h, and the levels of LAP*/LAP were determined (n = 3). (B) THP-1 cells were incubated in the absence or presence of PMA together with increasing doses of S6K1-inhibitor DG2 (S6K1-I), and LAP*/LAP protein amounts were measured (n = 3). Some of the control lanes without S6K1-I have been omitted (thin lines). (C) THP-1 cells were incubated with PMA in the absence/presence of the RSK inhibitor (RSK-I) BI-D1870 (2.5 μM), and LAP*/LAP and LIP levels were evaluated (n = 5). An additional control lane without PMA has been removed (thin line). (D) The LAP/LIP ratio of the experiment shown in C was calculated as described in Fig 1B. (E) MM-6 cells were incubated with RSK-I and/or PMA, and LAP*/LAP levels were measured (n = 4). (F) THP-1 cells were incubated ± PMA up to 18 h, and the levels of RSK and p-RSK were determined (n = 3). (G) THP-1 cells were incubated with the MEK inhibitor (MEK-I) U0126 (5 μM) or RSK-I and/or PMA for 24 h. The levels of LAP*/LAP, (p-)ERK1/2, (p-)RSK, and (p-)rpS6 were monitored (n = 5; arrows: phosphorylated and unphosphorylated rpS6). (H) HeLa cells were transfected with RSK1 siRNA (I: 50 nM, II: 100 nM) or control siRNA (Co; 100 nM), and following an incubation period of 24 h, the levels of LAP*/LAP and RSK1 were determined (n = 3). (I) THP-1 cells were incubated for 72 h with VitD3 together with increasing doses of RSK-I. Subsequently, LAP*/LAP protein amounts were assessed (n = 3). Lanes showing the effect of RSK-I on unstimulated cells have been removed (thin line).

Initiation of translation may be regulated by mTOR/S6K- or RSK-dependent signalling cascades [21]. Therefore, differentiating THP-1 cells were coincubated with the mTOR inhibitor rapamycin and with an inhibitor of S6K1 which is a substrate of mTOR [22]. Suitable doses and time frames for the application of the inhibitors were derived from the manufacturers’ instructions and further adapted by dose response and time course experiments. Both the mTOR inhibitor and the S6K1 inhibitor showed no effect on PMA-induced C/EBPβ expression in THP-1 cells (Fig 3A and 3B). In contrast, coincubation of cells with the RSK-inhibitor BI-D1870 (RSK-I; an ATP competitor targeting the N-terminal kinase domain of RSK) considerably reduced the PMA-induced LAP*/LAP production up to 24 h (Fig 3C and 3G) and virtually prevented an increase in the LAP/LIP ratio (at 12 h: -RSK-I: 10, +RSK-I: 2.5; Fig 3D). The effective dose of the applied RSK-I was identified in dose response experiments using PMA-treated THP-1 cells (S1 Fig).

Equivalent results were obtained using MM-6 cells (Fig 3E). In addition, the basically low levels of total and phosphorylated RSK can be dramatically increased in THP-1 by treatment with PMA up to 18 h (Fig 3F). Coincubation of cells with PMA and the MEK inhibitor U0126 (MEK-I) inhibited the PMA-induced production of LAP*/LAP, the phosphorylation of ERK1/2 (S217, Y221), and phosphorylation of RSK (T573) (Fig 3G). Since RSK-I only blocks RSK activity but not RSK phosphorylation by other kinases, RSK-I did not affect the levels of p-RSK (Fig 3G). Treatment with MEK-I or RSK-I also reduced the phosphorylation of rpS6 (Fig 3G) which is a substrate of RSK [23]. These data were supplemented by an siRNA approach against RSK1 which significantly reduced the expression of C/EBPβ-LAP*/LAP proteins (Fig 3H). The influence of RSK activity on LAP*/LAP formation was further validated in RSK-I dose response experiments using VitD3-treated (72 h) THP-1 cells (Fig 3I). These data suggest that during monocytic differentiation, the expression of the larger C/EBPβ isoforms is regulated by MEK/RSK-dependent signalling pathways.

### LAP*/LAP expression depends on phosphorylated eIF4B

**Fig 4:**
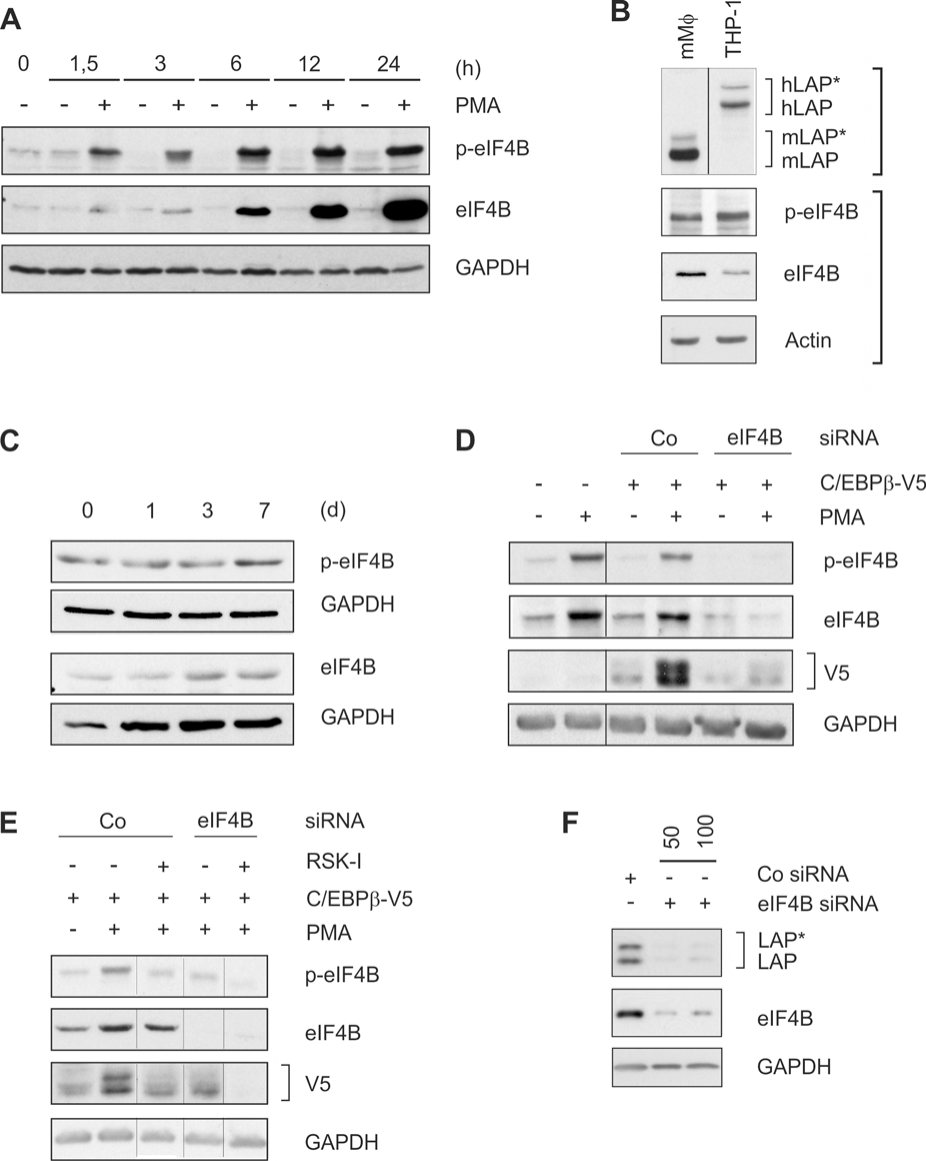
Phosphorylated eIF4B is required for enhanced C/EBPβ-LAP*/LAP expression. (A) THP-1 monocytic cells were incubated for 24 h ± PMA, and the amounts of (p-)eIF4B were detected (n = 3). (B) The levels of murine and human LAP*/LAP (murine: 38/34 kDa, human: 44/42 kDa) and (p-)eIF4B were determined in murine macrophage-like cells and PMA-treated (24 h) THP-1 cells (n = 4). Human/murine LAP*/LAP and (p-)eIF4B/Actin have been determined on different gels (indicated by square brackets). An additional negative control lane has been omitted from the upper gel (thin line). (C) Levels of (p-)eIF4B were measured in differentiating primary human monocytes up to 7 d (n = 3). (D) THP-1 cells were transfected for 24 h with 100 nM of a C/EBPβ-V5 expression plasmid and 25 nM eIF4B siRNA or control siRNA. Following an incubation with PMA for 3 h, protein levels of (p-)eIF4B and V5 were measured (n = 3). (E) Cells were treated as described in B and selected approaches were additionally treated with 2.5 μM RSK-I for 3 h (in parallel to the PMA treatment; n = 3). In D and E, additional control lanes without PMA have been removed to enhance the clarity of the panels (thin lines). (F) HeLa cells were transfected with control (100 nM) or 50 or 100 nM eIF4B siRNA. Following an incubation period of 24 h, protein levels of LAP*/LAP and eIF4B were determined (n = 3).

To connect MEK/RSK-signalling with protein synthesis, the level/phosphorylation status of specific RSK target proteins of the translation initiation machinery [23] was determined during monocytic differentiation. Under this condition, we observed a sustained increase in the expression and phosphorylation of the direct RSK target eIF4B from a low to a high level within 24 h of PMA treatment in THP-1 cells (Fig 4A). A prominent level of (p-)eIF4B was also found in murine macrophage-like cells which concomitantly exhibit high levels of C/EBPβ (Fig 4B) as well as in differentiating primary human monocytes (Fig 4C). Next, a C/EBPβ-V5 overexpression system was used to further establish the involvement of RSK-dependent eIF4B phosphorylation in LAP*/LAP production. Transfection of THP-1 cells led to a modest expression of V5-tagged C/EBPβ protein which was significantly enhanced by PMA treatment (Fig 4D). Both effects were remarkably reduced by eIF4B siRNA but not control siRNA (Fig 4D). Addition of RSK-I (which reduces phosphorylation but not the level of eIF4B) also inhibited C/EBPβ-V5 expression with a synergistic effect using both the inhibitor and siRNA (Fig 4E). In addition, siRNA against eIF4B inhibited the endogenous expression of the larger C/EBPβ isoforms in HeLa cells (Fig 4F). These data suggest that the helicase accessory protein eIF4B in its phosphorylated state is involved in the RSK-dependent expression of the larger C/EBPβ isoforms.

### Inhibition of PKR reduces the expression of (p-)eIF4B and LAP*/LAP

**Fig 5:**
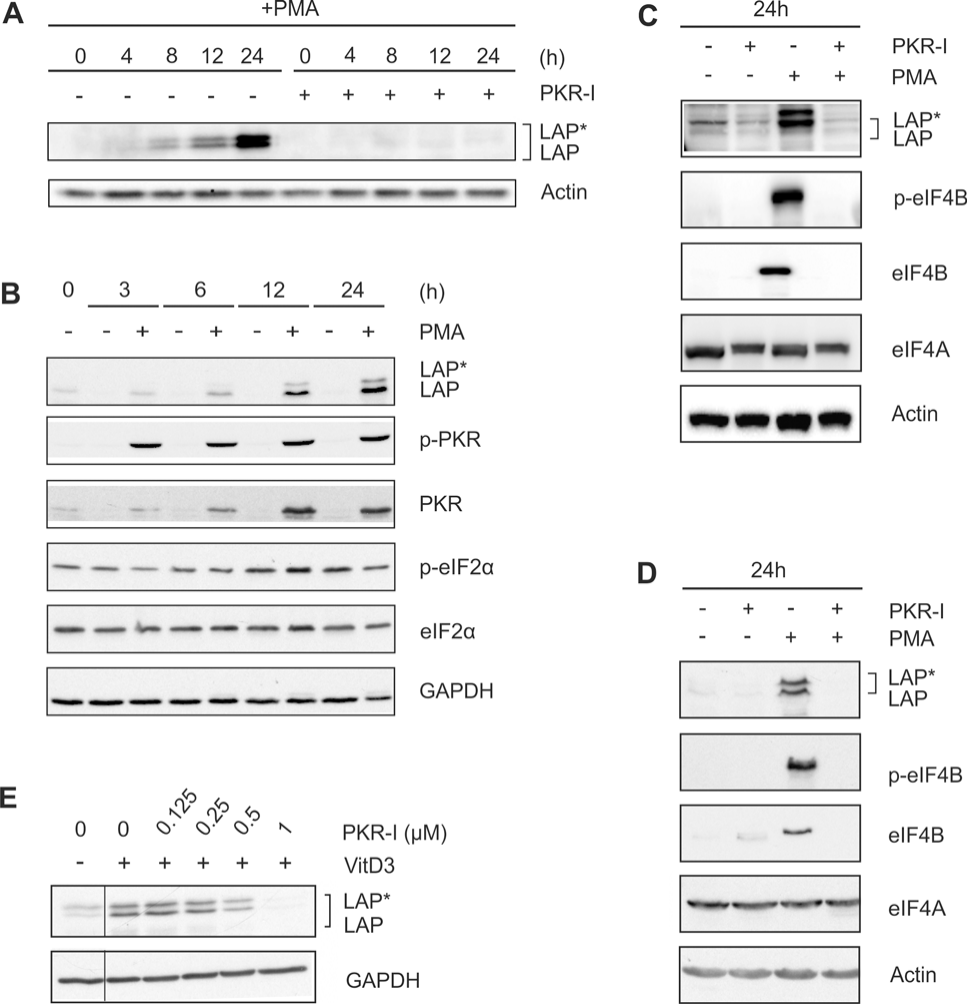
PKR inhibition prevents C/EBPβ and eIF4B expression. (A) THP-1 cells were incubated with PMA ± 1 μM PKR inhibitor (PKR-I) up to 24 h, and the levels of LAP*/LAP were determined (n = 3). (B) THP-1 cells were incubated ± PMA up to 24 h, and the levels of LAP*/LAP, (p-)PKR, and (p-)eIF2α were measured (n = 3). (C) THP-1 cells were exposed to PKR-I and/or PMA as indicated, and LAP*/LAP, (p-)eIF4B, and eIF4A levels were analysed (n = 3). (D) MM-6 cells were treated as indicated, and the levels of LAP*/LAP, (p-)eIF4B, and eIF4A were determined (n = 3). (E) THP-1 cells were incubated with VitD3 (72 h) and increasing doses of PKR-I and LAP*/LAP protein amounts were analysed (n = 3). Lanes showing the effect of PKR-I on unstimulated cells have been removed (thin line).

A PKR-dependent decrease in eIF2α activity has been shown earlier to mediate the expression of the larger C/EBPβ isoforms [5]. Treatment of cells with a specific PKR inhibitor (PKR-I) completely inhibited the differentiation-induced expression of LAP*/LAP (Fig 5A). The effective dose of the applied PKR-I was identified in dose response experiments using PMA-treated THP-1 cells (S2 Fig). Furthermore, following PMA treatment, we observed an increase in the level of PKR up to 24 h (Fig 5B). Phosphorylated PKR could be detected already at an early time point (3 h) of stimulation. Unexpectedly, the level of eIF2α and its degree of phosphorylation remained constant throughout the entire 24 h incubation period (Fig 5B). Treatment of cells with PKR-I also significantly inhibited the expression of (p-)eIF4B (Fig 5C). Essentially the same results were obtained using MM-6 cells (Fig 5D). VitD3-induced (72 h) LAP*/LAP expression in THP-1 cells was also inhibited by PKR-I application in a dose dependent manner (Fig 5E). In contrast, the amount of RNA helicase eIF4A was not affected in both THP-1 and MM-6 cells by treatment with PMA, MEK-I, RSK-I, or PKR-I (Fig 5C and 5D, data not shown). Our results suggest that PKR regulates LAP*/LAP production by mediating eIF4B expression in an eIF2α-independent manner.

### LAP*/LAP proteins are stabilised in models of monocytic differentiation

**Fig 6:**
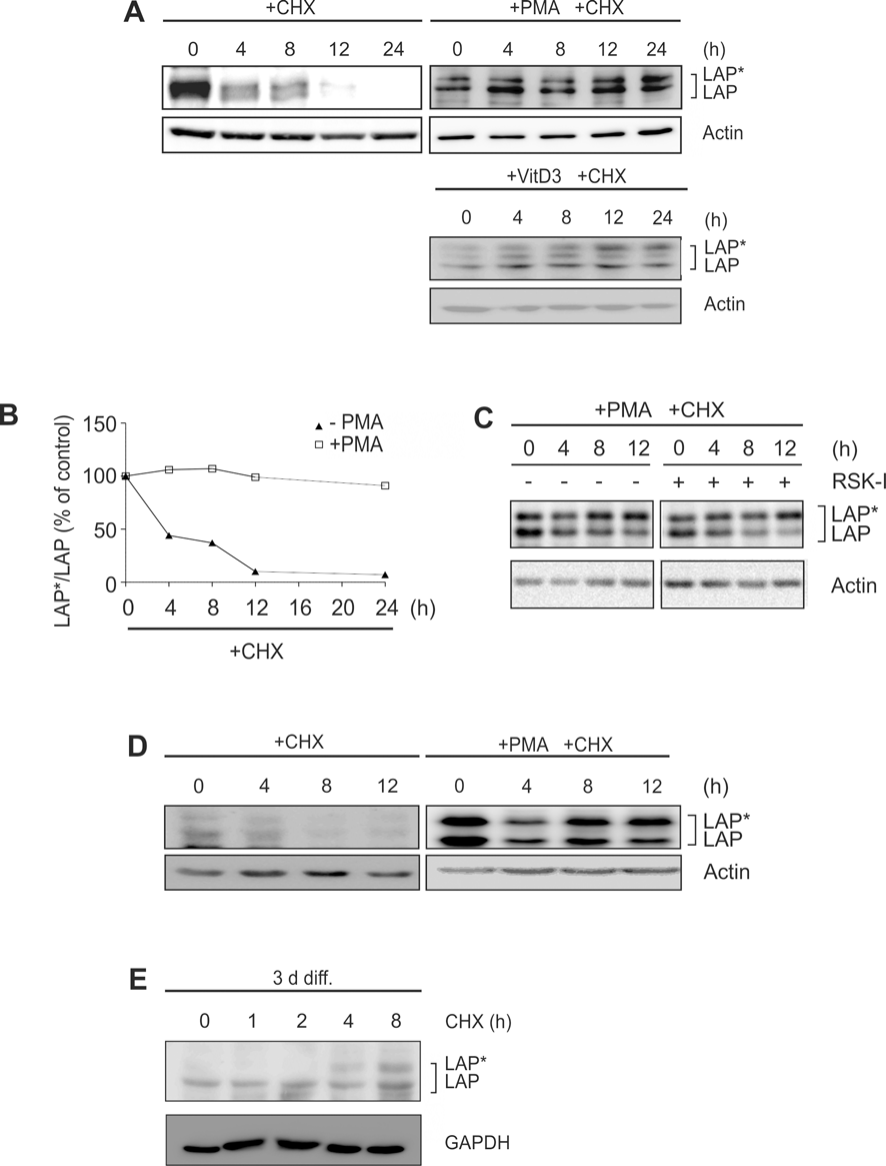
LAP*/LAP protein levels are stabilised in models of monocytic differentiation. (A) THP-1 monocytic cells were cultivated in the absence (left, 24 h) or presence of differentiating stimuli (PMA: 24 h; VitD3: 72 h). Then, cells were preincubated for 0.5 h with 10 μg/ml CHX before LAP*/LAP protein levels were analysed at the indicated time points (n = 3). To visualize the basically low LAP*/LAP levels in untreated cells, a prolonged exposure time and a more sensitive HRP substrate were applied (5 min Femto Plus *vs*. 1 min ECL). (B) Densitometric analysis of the experiment shown in A is depicted. The LAP*/LAP levels in THP-1 cells cultivated for 24 h in the absence or presence of PMA were defined as the respective 100% control. (C) THP-1 cells were incubated with PMA for 24 h. Following a 0.5 h treatment of cells with CHX ± RSK-I, the LAP*/LAP protein levels were analysed at the indicated time points (n = 3). (D) MM-6 cells were cultivated ± PMA for 24 h, and following addition of CHX, the LAP*/LAP levels were analysed at the indicated time points (n = 3). (E) Primary human monocytes were differentiated for 3 d (3 d diff.) and then preincubated for 0.5 h with CHX, before LAP*/LAP protein levels were analysed at the indicated time points (n = 3).

**Fig 7:**
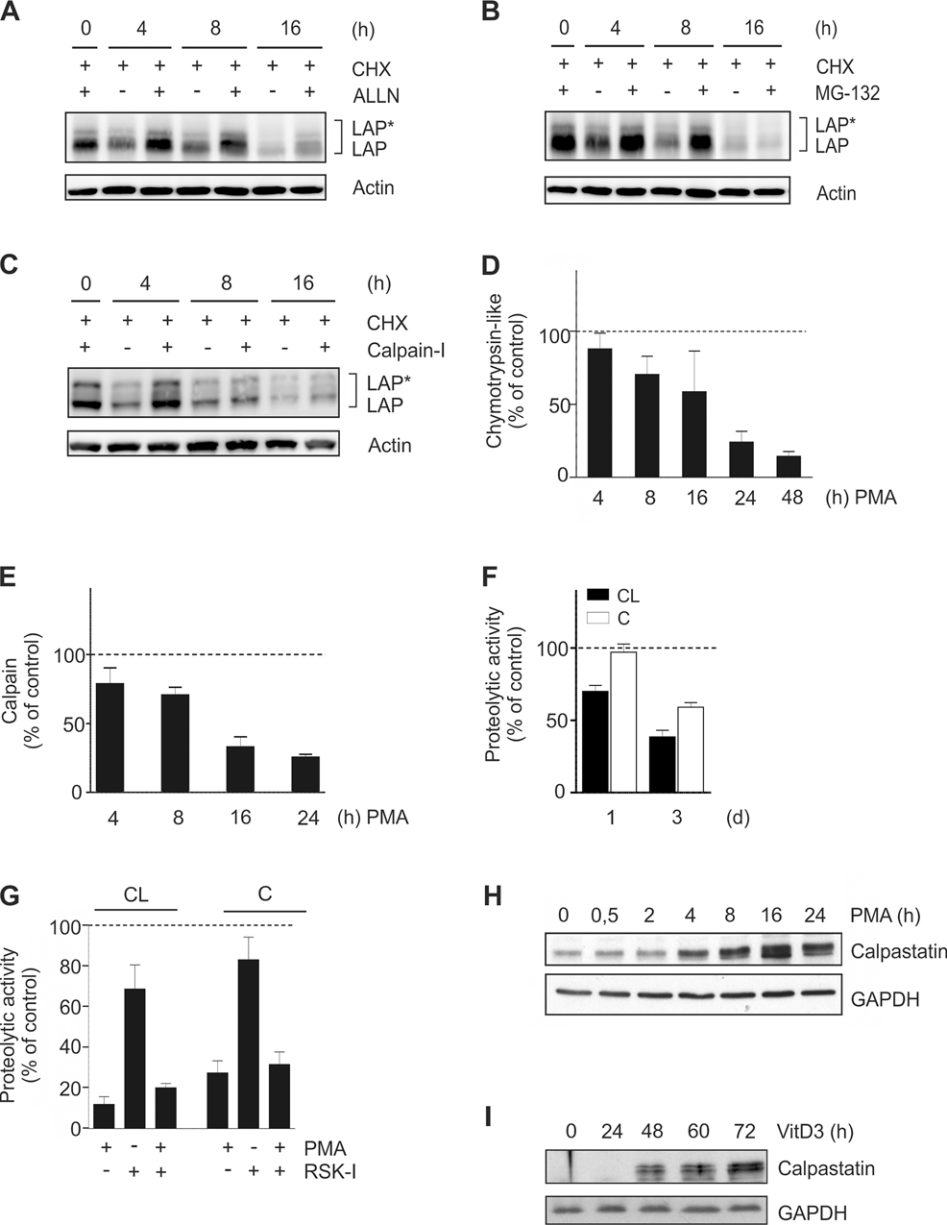
Inhibition of proteasome and calpain activities (A-C) THP-1 cells were treated with CHX ± 50 μM ALLN (A), 50 μM MG-132 (B), or 100 μM Calpain-inhibitor V (calpain-I; C). LAP*/LAP levels were detected at the indicated time points (n = 3 each). (D-E) THP-1 cells were treated with PMA up to 24 or 48 h and the chymotrypsin-like activity of the proteasome (D) as well as the calpain activity (E) were determined in whole cell extracts using protease-specific substrates (mean±SD; n≥3, duplicates). The respective proteolytic activity at 0 h was defined as 100% (dashed line). (F) Primary human monocytes were cultivated up to 3 d. The chymotrypsin-like (CL) and the calpain (C) proteolytic activities were determined at days 0, 1, and 3 (mean±SD; n = 2, representative for n = 4 experiments). The respective proteolytic activity at day 0 was defined as 100% (dashed line). (G) THP-1 cells were incubated for 24 h ± PMA in the absence or presence of RSK-I. The chymotrypsin-like (CL) proteasome and calpain (C) activities were measured as described above (mean±SD; n = 3, duplicates). (H-I) THP-1 cells were incubated with PMA up to 24 h (H) or with VitD3 up to 72 h (I) and the levels of calpastatin were determined at the indicated time points (n = 3).

In a second series of experiments we investigated whether posttranslational mechanisms contribute to C/EBPβ-LAP*/LAP expression during monocytic differentiation. Using the translation inhibitor CHX, the protein stability of LAP*/LAP was assessed in monocytic cells. In the absence of differentiation-inducing stimuli, the basically low LAP*/LAP levels were almost completely degraded at 12 h in THP-1 cells (please note that for detection of these very low levels a much longer exposure time and a more sensitive substrate were used in some experiments: see Fig 6A, Fig 7A–7C). Remarkably, no proteolysis of LAP*/LAP was observed in THP-1 cells following PMA stimulation for 24 h (half-life ≥24 h; Fig 6A and 6B) or following exposure to VitD3 for 72 h (Fig 6A). These results were confirmed in PMA-treated THP-1 cells by pulse-chase experiments with ^35^-labelled C/EBPβ proteins (data not shown). Under this condition, treatment of cells with RSK-I had no effect on the stability of LAP*/LAP in differentiating THP-1 cells (Fig 6C). A significant stabilization of LAP*/LAP proteins was also observed in MM-6 cells in the presence of PMA (Fig 6D), differentiating primary human monocytes (Fig 6E), and murine macrophage-like cells (data not shown). These data suggest a marked (but RSK-independent) stabilization of C/EBPβ-LAP*/LAP proteins during monocytic differentiation.

### Chymotrypsin-like proteasomal and calpain activities are specifically inhibited

Next the influence of different proteolytic activities on C/EBPβ protein stability was assessed. In unstimulated THP-1 cells, the application of ALLN (a substance inhibiting the proteolytic activities of the proteasome, calpain 1 and 2, cathepsin B and L, and neutral cysteine proteases) temporarily stabilizes the amount of detectable LAP*/LAP in comparison to the ALLN-free controls (Fig 7A). To further identify the proteases involved in C/EBPβ degradation, more specific inhibitors were used either inhibiting the proteasome (MG-132), calpain (Calpain-Inhibitor V), or cathepsin (Cathepsin Inhibitor I) proteases. As shown by elevated C/EBPβ protein levels within 4 to 8 h in the presence of the respective inhibitors, both the proteasome and calpain activities appear to be involved in C/EBPβ degradation (Fig 7B and 7C), whereas no influence of cathepsins could be detected (data not shown). These data indicate an involvement of proteasome and calpain protease systems in the degradation of C/EBPβ isoforms. To elucidate whether the identified C/EBPβ degrading proteolytic activities are modulated during monocytic differentiation, the specific proteolytic activities were analysed in whole cell extracts of PMA-treated THP-1 cells. These experiments showed a significant inhibition of the chymotrypsin-like proteolytic activity of the proteasome by PMA with a maximal effect at 48 h (Fig 7D). The trypsin-like activity of the proteasome was only slightly affected at 8 h and no PMA effect was seen on the caspase-like activity (S3 Fig, data not shown). We also detected a strong inhibitory effect of PMA on the calpain activity with a maximal reduction at 24 h (Fig 7E). In addition, a further decrease of the chymotrypsin-like and the calpain proteolytic activities was observed in primary human monocytes cultivated up to 3 d (Fig 7F). The activity of cathepsins, however, was not affected (data not shown). Consistent with the results shown in the previous figure, coincubation with the RSK inhibitor had no significant effect on PMA-mediated inhibition of the chymotrypsin-like proteasomal or the calpain activity (Fig 7G). Since these experiments (using protease-specific labelled substrates) suggest that protease activities are directly affected, we monitored the expression of calpastatin, one of the most powerful regulators of the calpain system [24]. We found a continuous increase in the level of endogenous calpastatin in PMA- and VitD3-differentiated THP-1 cells (Fig 7H and 7I) which suggests that this inhibitor protein contributes at least in part to the observed inhibition of calpain activity.

## Discussion

Using different models for (pre)monocytic differentiation we demonstrated a marked increase in the larger C/EBPβ isoforms LAP*/LAP and a dramatic increase in the LAP/LIP ratio consistent with earlier results [17]. It has been shown that the larger C/EPBβ isoforms modulate mechanisms inhibiting proliferation, thereby participating in differentiation, and orchestrate gene expression patterns of effector molecules involved in fundamental monocyte functions [1, 2, 17]. The strong elevation of LAP*/LAP protein under differentiation-supporting conditions was accompanied by an only modest and somewhat retarded increase in mRNA suggesting that (post)translational mechanisms play a key role in mediating this protein expression. In primary human monocytes significant levels of LAP*/LAP protein were detected which only slightly further increased during ongoing differentiation up to 7 d, similar as described [3]. Therefore, to characterize the mechanisms which regulate the massive increase in LAP*/LAP, we focused in the majority of the presented experiments on premonocytic models of monocyte differentiation.

Our experiments revealed a marked increase in the levels of total and phosphorylated RSK in monocytic differentiation models. Furthermore, we showed that the prominent elevation of LAP*/LAP during this state of monocytic development was mediated *via* the MEK/ERK/RSK cascade. RSK is a MAPK involved in the regulation of protein translation by activating rpS6 and eIF4B [23]. Several MAPK including RSK have been suggested to be involved in the differentiation of several cell types including monocytic cells [25]. Alternative translation of C/EBPβ towards the small isoform LIP depends on mTOR which activates eIF4E [5] and phosphorylates S6K1 [23]. For instance, during early myelomonocytic differentiation, the FLT3-induced LIP production has been shown to be mediated by mTOR [15]. Our data demonstrated that mTOR/S6K1 were not involved in PMA-induced monocytic LAP*/LAP expression. However, this shift towards LAP*/LAP was accompanied by rpS6 phosphorylation which was sensitive to RSK and MEK inhibition. A cluster of conserved rpS6 serine residues can be phosphorylated by RSK family members [23]. rpS6 has been described as a structural protein of the 40S ribosomal subunit interacting with essential components of the translation initiation complex [23, 26, 27]. Interestingly, it has been proposed that rpS6 may be involved in the translation of mRNA species containing 5’ terminal oligopyrimidine tracts [28], a structure also characteristic for C/EBPβ mRNA. Phosphorylated rpS6 has been reported to be involved in the control of cell size [29] and terminal differentiation of muscle cells [30].

Our data demonstrated significant levels of phosphorylated eIF4B in different models of monocytic differentiation. Most important, we found that the production of LAP*/LAP protein under the applied conditions depends on phosphorylated eIF4B. During initiation of translation the mRNA is bound by the heterotrimeric eIF4F protein complex consisting of cap-binding factor eIF4E, the scaffold protein eIF4G, and the ATP-dependent RNA helicase eIF4A [31]. eIF4A facilitates the processing and unwinding of mRNAs containing more complex 5´UTR secondary structures to enable binding of the preinitiation complex (PIC). The activity of eIF4A is significantly enhanced by accessory proteins such as eIF4B and eIF4H [21]. Both proteins support translation by promoting a unidirectional movement of eIF4A and enhancing its ATP binding activity as well as mRNA affinity [27]. It has also been proposed that eIF4B contributes to the association of the eIF4G-mRNA complex with the 40S subunit of the ribosome [21]. Recently, it has been shown that eIF4A-associated translational regulation may play a role during myogenic differentiation [32]. In our study, the levels of eIF4A remained stable under the applied conditions. The intracellular amount of eIF4A has been shown to exceed the levels of its binding partners [33] which indicates that translational events are not directly regulated by this translation factor. Thus, the activation of eIF4A by rate-limiting eIF4B may be a crucial step in PIC recruitment under certain cellular conditions [33]. With respect to the high GC content in the 5’UTR of the C/EBPβ mRNA (77%) [2] and the predicted formation of stable stem loop structures [8], an involvement of further proteins in the initiation of C/EBPβ mRNA translation appears probable, such as RNA helicase eIF4A and its accessory factors, e.g. eIF4B.

In addition, our results suggest that PKR-dependent pathways significantly contribute to the expression of LAP*/LAP in differentiating monocytic cells. PKR, initially described as an antiviral kinase activated by dsRNA, is able to reduce general protein translation by inhibitory phosphorylation of eIF2α [34]. A PKR-dependent reduction of eIF2α activity is associated with a predominant expression of the larger C/EBPβ isoforms under certain conditions, whereas an increased eIF2α activity favours the formation of LIP [5]. Our results showed an increase in (p-)PKR but no change in the level or phosphorylation of eIF2α. However, coincubation with a PKR inhibitor markedly reduced the PMA-induced increase of (p-)eIF4B. Several reports describe eIF2α-independent alternative functions of PKR [35]. It has also been described earlier that PMA under certain conditions has no effect on eIF2α phosphorylation [36]. Our data suggest that PKR is involved in the regulation of LAP*/LAP expression by controlling eIF4B expression in an eIF2α-independent manner. Further studies are necessary to dissect these here described new alternative functionalities of PKR.

We also found a marked stabilization of C/EBPβ-LAP*/LAP proteins in monocytic differentiation models. The dependency of C/EBPβ isoform half-life on certain cellular conditions has been shown before. For instance, it has been reported that in proliferating cells, e.g. fibroblasts and myotubes, LAP*/LAP exhibit a half-life of 1–4 h [12, 37, 38]. Under differentiation-supporting conditions, the half-life appears to be considerably prolonged in preadipocytes (8–29 h) [10, 39, 40]. Additional conditions enhancing C/EBPβ protein half-life have also been described, e.g. transfection of kinases targeting tyrosine 79 (>6 h) [41] or suppression of C/EBPβ SUMOylation (>12 h) [38, 40].

In addition, the degradation of LAP*/LAP could be reduced in unstimulated cells by proteasome or calpain inhibitors. This is in good agreement with earlier results describing a degradation of C/EBPβ proteins by the proteasome or calpain [10–12]. Proteasome-associated degradation of C/EBPβ has also been reported to negatively regulate the expression of certain C/EBPβ target genes such as elastin [42]. Consistently, we observed an inhibition of the chymotrypsin-like proteolytic activity of the proteasome as well as calpain activity in differentiating THP-1 cells. A further reduction of both proteolytic activities was also observed in differentiating primary human monocytes. Since we used protease assays including specific fluorescence-labelled artificial substrates, these data suggest that under the applied cellular conditions, proteasome and calpain activities are directly affected. The entire 26S proteasome consists of two subcomplexes (19S regulatory particle, 20S core particle) and at least 33 different subunits [43] enabling the formation of standard as well as immunoproteasomes with different specificities [44]. The activity of the proteasome can be regulated by different signalling molecules and pathways. For instance, the MAPKKK family protein apoptosis signal-regulating kinase (ASK1) negatively regulates the proteasome by inhibiting the ATPase of the 19S particle [45]. TGF-β- and SMAD3/p53-dependent signalling has been described to inhibit the 20S proteasome activity by repressing the proteasome activator subunit 3 (PSME3) [46]. Calpains comprise a family of calcium-dependent cysteine proteases, e.g. the heterodimer μ- and m-calpain consisting of a catalytic and a regulatory subunit which catalyse limited proteolysis of a variety of targets [24]. The proteolytic activity of calpain is negatively regulated by its endogenous inhibitor calpastatin [24]. Our analyses showed a considerable increase in calpastatin expression following PMA stimulation correlating with both the decreased calpain proteolytic activity and the enhanced LAP*/LAP levels. This suggests that during monocytic differentiation, the inhibition of calpains may be driven at least in part by increasing calpastatin amounts. A differentiation-supporting role of calpastatin has been described in adipocytes and neuronal stem cells [47, 48]. Further reports demonstrate that calpain can be activated by a conformational change induced by calcium binding in response to calcium signalling [24]. Phosphorylation of calpains by PKC, ERK, or PKA has also been shown to increase or decrease calpain activity [24].

In addition, several reports suggest further regulatory mechanisms which directly influence the stability of the C/EBPβ protein. For instance, multiubiquitination and proteasomal degradation of C/EBPβ has been described [10] which has been increased by previous SUMOylation [38, 40]. Homo/heterodimerization has been demonstrated as an additional mechanism to protect C/EBPβ monomers from proteasome-associated degradation [49]. However, it is still controversial whether C/EBPβ is multiubiquitinated [49] and whether the proteasome is directly involved in C/EBPβ cleavage [13]. Moreover, phosphorylation of murine C/EBPβ at threonine 188 has been shown to be protective especially against μ-calpain-dependent degradation [39]. In human cells, phosphorylation at tyrosine 79 enhances C/EBPβ protein stability [41], whereas phosphorylation at threonine 235 supports proteolysis [50].

The importance of the regulation of the translational machinery during cellular differentiation has been recognised earlier [31], also with respect to maturation of monocytic cells [51]. Furthermore, proteolysis-associated processes, e.g. involving proteasomes or calpains, have been proposed as important regulatory mechanisms controlling cellular development [52, 53], including differentiation of hematopoietic cells [54]. In comparison to transcriptional regulation, translational control of gene expression together with proteolytic fine-tuning allows a faster change in protein concentrations [31]. Furthermore, it has been proposed that this immediate interaction represents an important quality control of newly synthesised proteins at the ribosomes [55]. In the present study, we show that translation- and proteolysis-associated mechanisms based on only limited mRNA changes induce a shift in C/EBPβ expression towards LAP*/LAP resulting in a dramatically increased protein expression in models of monocytic differentiation. Under this condition, MEK/RSK-mediated LAP*/LAP formation depends on phosphorylated eIF4B and rpS6 which may form a functional clamp within the PIC/40S ribosome to guide the process of translation initiation. Our experiments suggest new eIF2α-independent functions of PKR potentially establishing additional regulatory avenues for this kinase. The switch from mTOR-induced LIP formation during early myelomonocytic differentiation to RSK-mediated signalling in further developed monocytic cells to orchestrate LAP*/LAP production, pushed by an increased protein stability, may be a prototypical example for the regulation of protein expression during selected processes of differentiation/proliferation and cell homeostasis. Thus, this study may contribute to a better understanding of proliferation/differentiation-related diseases such as sepsis or leukemia.

## Supporting Information

S1 Fig Determination of the effective dose of the applied RSK-I. THP-1 cells were incubated for 24 h with PMA together with increasing doses of RSK-I. Subsequently, LAP*/LAP protein amounts were assessed (n = 3).
(PDF)

S2 Fig Determination of the effective dose of the applied PKR-I. THP-1 cells were incubated for 24 h with PMA together with increasing doses of PKR-I. Subsequently, LAP*/LAP protein amounts were assessed (n = 3). Additional lanes showing the effect of PKR-I on unstimulated cells have been removed (thin lines).
(PDF)

S3 Fig Trypsin-like proteolytic activity in differentiating THP-1 cells. THP-1 cells were treated with PMA up to 24 h and the trypsin-like activity of the proteasome was determined in whole cell extracts using protease-specific substrates (mean±SD; n = 3, duplicates).
(PDF)

## References

[1] Ramji DP, Foka P. CCAAT/enhancer-binding proteins: structure, function and regulation. Biochem J. 2002; 365:561–75. . 12006103 10.1042/BJ20020508 PMC1222736

[2] Huber R, Pietsch D, Panterodt T, Brand K. Regulation of C/EBPbeta and resulting functions in cells of the monocytic lineage. Cell Signal. 2012; 24(1):1287–96. . 22374303 10.1016/j.cellsig.2012.02.007

[3] Pham TH, Langmann S, Schwarzfischer L, El Chartouni C, Lichtinger M, Klug M, et al CCAAT enhancer-binding protein beta regulates constitutive gene expression during late stages of monocyte to macrophage differentiation. J Biol Chem. 2007; 282(30):21924–33. . 17540774 10.1074/jbc.M611618200

[4] Zwergal A, Quirling M, Saugel B, Huth KC, Sydlik C, Poli V, et al C/EBP beta blocks p65 phosphorylation and thereby NF-kappa B-mediated transcription in TNF-tolerant cells. J Immunol. 2006; 177(1):665–72. . 16785565 10.4049/jimmunol.177.1.665

[5] Calkhoven CF, Muller C, Leutz A. Translational control of C/EBPalpha and C/EBPbeta isoform expression. Genes Dev. 2000; 14(15):1920–32. . 10921906 PMC316813

[6] Wethmar K, Smink JJ, Leutz A. Upstream open reading frames: molecular switches in (patho)physiology. Bioessays. 2010; 32(10):885–93. 10.1002/bies.201000037 20726009 PMC3045505

[7] Timchenko NA, Wang GL, Timchenko LT. RNA CUG-binding protein 1 increases translation of 20-kDa isoform of CCAAT/enhancer-binding protein beta by interacting with the alpha and beta subunits of eukaryotic initiation translation factor 2. J Biol Chem. 2005; 280(21):20549–57. . 15788409 10.1074/jbc.M409563200

[8] Timchenko LT, Iakova P, Welm AL, Cai ZJ, Timchenko NA. Calreticulin interacts with C/EBPalpha and C/EBPbeta mRNAs and represses translation of C/EBP proteins. Mol Cell Biol. 2002; 22(20):7242–57. Epub 2002/09/21. 12242300 10.1128/MCB.22.20.7242-7257.2002 PMC139801

[9] Bergalet J, Fawal M, Lopez C, Desjobert C, Lamant L, Delsol G, et al HuR-mediated control of C/EBPbeta mRNA stability and translation in ALK-positive anaplastic large cell lymphomas. Mol Cancer Res. 2011; 9(4):485–96. 10.1158/1541-7786.MCR-10-0351 21343335

[10] Lechner S, Mitterberger MC, Mattesich M, Zwerschke W. Role of C/EBPbeta-LAP and C/EBPbeta-LIP in early adipogenic differentiation of human white adipose-derived progenitors and at later stages in immature adipocytes. Differentiation. 2013; 85(1–2):20–31. Epub 2013/01/15. 10.1016/j.diff.2012.11.001 . 23314288

[11] Li Y, Bevilacqua E, Chiribau CB, Majumder M, Wang C, Croniger CM, et al Differential control of the CCAAT/enhancer-binding protein beta (C/EBPbeta) products liver-enriched transcriptional activating protein (LAP) and liver-enriched transcriptional inhibitory protein (LIP) and the regulation of gene expression during the response to endoplasmic reticulum stress. J Biol Chem. 2008; 283(33):22443–56. 10.1074/jbc.M801046200 18550528 PMC2504880

[12] Wei W, Yang H, Cao P, Menconi M, Chamberlain C, Petkova V, et al Degradation of C/EBPbeta in cultured myotubes is calpain-dependent. J Cell Physiol. 2006; 208(2):386–98. . 16646084 10.1002/jcp.20684

[13] Welm AL, Timchenko NA, Darlington GJ. C/EBPalpha regulates generation of C/EBPbeta isoforms through activation of specific proteolytic cleavage. Mol Cell Biol. 1999; 19(3):1695–704. . 10022857 10.1128/mcb.19.3.1695 PMC83963

[14] Baer M, Johnson PF. Generation of truncated C/EBPbeta isoforms by in vitro proteolysis. J Biol Chem. 2000; 275(34):26582–90. . 10856306 10.1074/jbc.M004268200

[15] Haas SC, Huber R, Gutsch R, Kandemir JD, Cappello C, Krauter J, et al ITD- and FL-induced FLT3 signal transduction leads to increased C/EBPbeta-LIP expression and LIP/LAP ratio by different signalling modules. Br J Haematol. 2010; 148(5):777–90. 10.1111/j.1365-2141.2009.08012.x 19958352

[16] Stirewalt DL, Radich JP. The role of FLT3 in haematopoietic malignancies. Nat Rev Cancer. 2003; 3(9):650–65. . 12951584 10.1038/nrc1169

[17] Gutsch R, Kandemir JD, Pietsch D, Cappello C, Meyer J, Simanowski K, et al CCAAT/enhancer-binding protein beta inhibits proliferation in monocytic cells by affecting the retinoblastoma protein/E2F/cyclin E pathway but is not directly required for macrophage morphology. J Biol Chem. 2011; 286(26):22716–29. 10.1074/jbc.M110.152538 21558273 PMC3123039

[18] Gorgoni B, Maritano D, Marthyn P, Righi M, Poli V. C/EBP beta gene inactivation causes both impaired and enhanced gene expression and inverse regulation of IL-12 p40 and p35 mRNAs in macrophages. J Immunol. 2002; 168(8):4055–62. . 11937564 10.4049/jimmunol.168.8.4055

[19] Brand K, Mackman N, Curtiss LK. Interferon-gamma inhibits macrophage apolipoprotein E production by posttranslational mechanisms. J Clin Invest. 1993; 91(5):2031–9. Epub 1993/05/01. 10.1172/JCI116425 8486772 PMC288201

[20] Gunther J, Vogt N, Hampel K, Bikker R, Page S, Muller B, et al Identification of two forms of TNF tolerance in human monocytes: differential inhibition of NF-kappaB/AP-1- and PP1-associated signaling. J Immunol. 2014; 192(7):3143–55. Epub 2014/02/28. 10.4049/jimmunol.1301610 . 24574500

[21] Parsyan A, Svitkin Y, Shahbazian D, Gkogkas C, Lasko P, Merrick WC, et al mRNA helicases: the tacticians of translational control. Nat Rev Mol Cell Biol. 2011; 12(4):235–45. Epub 2011/03/24. 10.1038/nrm3083 . 21427765

[22] Zoncu R, Efeyan A, Sabatini DM. mTOR: from growth signal integration to cancer, diabetes and ageing. Nat Rev Mol Cell Biol. 2011; 12(1):21–35. Epub 2010/12/16. 10.1038/nrm3025 21157483 PMC3390257

[23] Anjum R, Blenis J. The RSK family of kinases: emerging roles in cellular signalling. Nat Rev Mol Cell Biol. 2008; 9(10):747–58. 10.1038/nrm2509 18813292

[24] Storr SJ, Carragher NO, Frame MC, Parr T, Martin SG. The calpain system and cancer. Nat Rev Cancer. 2011; 11(5):364–74. Epub 2011/04/22. 10.1038/nrc3050 . 21508973

[25] Wang X, Studzinski GP. Raf-1 signaling is required for the later stages of 1,25-dihydroxyvitamin D3-induced differentiation of HL60 cells but is not mediated by the MEK/ERK module. J Cell Physiol. 2006; 209(2):253–60. . 16883571 10.1002/jcp.20731 PMC2814417

[26] Ruvinsky I, Meyuhas O. Ribosomal protein S6 phosphorylation: from protein synthesis to cell size. Trends Biochem Sci. 2006; 31(6):342–8. Epub 2006/05/09. 10.1016/j.tibs.2006.04.003 . 16679021

[27] Jackson RJ, Hellen CU, Pestova TV. The mechanism of eukaryotic translation initiation and principles of its regulation. Nat Rev Mol Cell Biol. 2010; 11(2):113–27. Epub 2010/01/23. 10.1038/nrm2838 . 20094052 PMC4461372

[28] Hagner PR, Mazan-Mamczarz K, Dai B, Balzer EM, Corl S, Martin SS, et al Ribosomal protein S6 is highly expressed in non-Hodgkin lymphoma and associates with mRNA containing a 5' terminal oligopyrimidine tract. Oncogene. 2011; 30(13):1531–41. Epub 2010/11/26. 10.1038/onc.2010.533 21102526 PMC3227680

[29] Ruvinsky I, Katz M, Dreazen A, Gielchinsky Y, Saada A, Freedman N, et al Mice deficient in ribosomal protein S6 phosphorylation suffer from muscle weakness that reflects a growth defect and energy deficit. PLoS One. 2009; 4(5):e5618 Epub 2009/05/30. 10.1371/journal.pone.0005618 19479038 PMC2682700

[30] Csibi A, Cornille K, Leibovitch MP, Poupon A, Tintignac LA, Sanchez AM, et al The translation regulatory subunit eIF3f controls the kinase-dependent mTOR signaling required for muscle differentiation and hypertrophy in mouse. PLoS One. 2010; 5(2):e8994 Epub 2010/02/04. 10.1371/journal.pone.0008994 20126553 PMC2813880

[31] Sonenberg N, Hinnebusch AG. Regulation of translation initiation in eukaryotes: mechanisms and biological targets. Cell. 2009; 136(4):731–45. Epub 2009/02/26. 10.1016/j.cell.2009.01.042 19239892 PMC3610329

[32] Galicia-Vazquez G, Di Marco S, Lian XJ, Ma JF, Gallouzi IE, Pelletier J. Regulation of eukaryotic initiation factor 4AII by MyoD during murine myogenic cell differentiation. PLoS One. 2014; 9(1):e87237 Epub 2014/01/28. 10.1371/journal.pone.0087237 24466343 PMC3900710

[33] Aitken CE, Lorsch JR. A mechanistic overview of translation initiation in eukaryotes. Nat Struct Mol Biol. 2012; 19(6):568–76. Epub 2012/06/06. 10.1038/nsmb.2303 . 22664984

[34] Taylor SS, Haste NM, Ghosh G. PKR and eIF2alpha: integration of kinase dimerization, activation, and substrate docking. Cell. 2005; 122(6):823–5. Epub 2005/09/24. 10.1016/j.cell.2005.09.007 . 16179248

[35] Donnelly N, Gorman AM, Gupta S, Samali A. The eIF2alpha kinases: their structures and functions. Cell Mol Life Sci. 2013; 70(19):3493–511. Epub 2013/01/29. 10.1007/s00018-012-1252-6 . 23354059 PMC11113696

[36] Grolleau A, Kaplan MJ, Hanash SM, Beretta L, Richardson B. Impaired translational response and increased protein kinase PKR expression in T cells from lupus patients. J Clin Invest. 2000; 106(12):1561–8. Epub 2000/12/20. 10.1172/JCI9352 11120763 PMC381471

[37] Sears RC, Sealy L. Multiple forms of C/EBP beta bind the EFII enhancer sequence in the Rous sarcoma virus long terminal repeat. Mol Cell Biol. 1994; 14(7):4855–71. . 8007984 10.1128/mcb.14.7.4855-4871.1994 PMC358858

[38] Chung SS, Ahn BY, Kim M, Choi HH, Park HS, Kang S, et al Control of adipogenesis by the SUMO-specific protease SENP2. Mol Cell Biol. 2010; 30(9):2135–46. Epub 2010/03/03. 10.1128/MCB.00852-09 20194620 PMC2863577

[39] Zhang YY, Li SF, Qian SW, Liu Y, Tang QQ, Li X. Phosphorylation prevents C/EBPbeta from the calpain-dependent degradation. Biochem Biophys Res Commun. 2012; 419(3):550–5. Epub 2012/03/01. 10.1016/j.bbrc.2012.02.058 . 22369944

[40] Liu Y, Zhang YD, Guo L, Huang HY, Zhu H, Huang JX, et al Protein inhibitor of activated STAT 1 (PIAS1) is identified as the SUMO E3 ligase of CCAAT/enhancer-binding protein beta (C/EBPbeta) during adipogenesis. Mol Cell Biol. 2013; 33(22):4606–17. Epub 2013/09/26. 10.1128/MCB.00723-13 24061474 PMC3838193

[41] Li X, Liu X, Wang G, Zhu X, Qu X, Yang Y, et al Non-receptor tyrosine kinases c-Abl and Arg regulate the activity of C/EBPbeta. J Mol Biol. 2009; 391(4):729–43. Epub 2009/07/01. 10.1016/j.jmb.2009.06.055 . 19563810

[42] Kuang PP, Goldstein RH. Regulation of elastin gene transcription by proteasome dysfunction. Am J Physiol Cell Physiol. 2005; 289(3):C766–73. Epub 2005/04/09. 10.1152/ajpcell.00525.2004 . 15814588

[43] Kunjappu MJ, Hochstrasser M. Assembly of the 20S proteasome. Biochim Biophys Acta. 2014; 1843(1):2–12. Epub 2013/03/20. 10.1016/j.bbamcr.2013.03.008 23507199 PMC3752329

[44] Kish-Trier E, Hill CP. Structural biology of the proteasome. Annu Rev Biophys. 2013; 42:29–49. Epub 2013/02/19. 10.1146/annurev-biophys-083012-130417 . 23414347 PMC4878838

[45] Um JW, Im E, Park J, Oh Y, Min B, Lee HJ, et al ASK1 negatively regulates the 26 S proteasome. J Biol Chem. 2010; 285(47):36434–46. Epub 2010/09/17. 10.1074/jbc.M110.133777 20843792 PMC2978573

[46] Ali A, Wang Z, Fu J, Ji L, Liu J, Li L, et al Differential regulation of the REGgamma-proteasome pathway by p53/TGF-beta signalling and mutant p53 in cancer cells. Nat Commun. 2013; 4:2667 Epub 2013/10/26. 10.1038/ncomms3667 24157709 PMC3876931

[47] Yajima Y, Sato M, Sorimachi H, Inomata M, Maki M, Kawashima S. Calpain system regulates the differentiation of adult primitive mesenchymal ST-13 adipocytes. Endocrinology. 2006; 147(10):4811–9. Epub 2006/07/22. 10.1210/en.2005-1647 . 16857754

[48] Santos DM, Xavier JM, Morgado AL, Sola S, Rodrigues CM. Distinct regulatory functions of calpain 1 and 2 during neural stem cell self-renewal and differentiation. PLoS One. 2012; 7(3):e33468 Epub 2012/03/21. 10.1371/journal.pone.0033468 22432027 PMC3303840

[49] Hattori T, Ohoka N, Inoue Y, Hayashi H, Onozaki K. C/EBP family transcription factors are degraded by the proteasome but stabilized by forming dimer. Oncogene. 2003; 22(9):1273–80. . 12618752 10.1038/sj.onc.1206204

[50] Atwood AA, Sealy L. Regulation of C/EBPbeta1 by Ras in mammary epithelial cells and the role of C/EBPbeta1 in oncogene-induced senescence. Oncogene. 2010; 29(45):6004–15. Epub 2010/09/08. 10.1038/onc.2010.336 20818427 PMC2978746

[51] Rosenbauer F, Tenen DG. Transcription factors in myeloid development: balancing differentiation with transformation. Nat Rev Immunol. 2007; 7(2):105–17. . 17259967 10.1038/nri2024

[52] Yajima Y, Kawashima S. Calpain function in the differentiation of mesenchymal stem cells. Biol Chem. 2002; 383(5):757–64. Epub 2002/07/11. 10.1515/BC.2002.079 . 12108540

[53] Konstantinova IM, Tsimokha AS, Mittenberg AG. Role of proteasomes in cellular regulation. Int Rev Cell Mol Biol. 2008; 267:59–124. Epub 2008/06/12. 10.1016/S1937-6448(08)00602-3 . 18544497

[54] Liu YC. Ubiquitin ligases and the immune response. Annu Rev Immunol. 2004; 22:81–127. Epub 2004/03/23. 10.1146/annurev.immunol.22.012703.104813 . 15032575

[55] Rodrigo-Brenni MC, Hegde RS. Design principles of protein biosynthesis-coupled quality control. Dev Cell. 2012; 23(5):896–907. Epub 2012/11/17. 10.1016/j.devcel.2012.10.012 . 23153486

