# Supplementary material for: C/EBPβ-LAP*/LAP Expression Is Mediated by RSK/eIF4B-Dependent Signalling and Boosted by Increased Protein Stability in Models of Monocytic Differentiation
Source: PLoS One. 2015 Dec 8;10(12):e0144338. doi: 10.1371/journal.pone.0144338 (PMC4672875; doi:10.1371/journal.pone.0144338)
Supplement: S1 Fig — THP-1 cells were incubated for 24 h with PMA together with increasing doses of RSK-I. Subsequently, LAP*/LAP protein amounts were assessed (n = 3). (PDF) [file pone.0144338.s001.pdf]

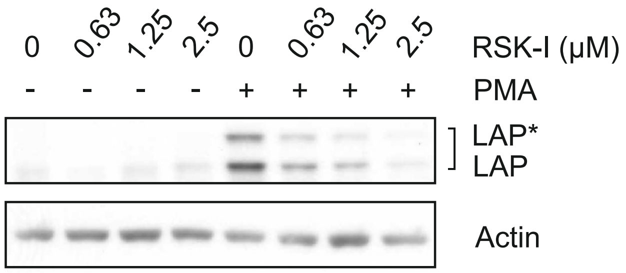

S1 Figure. Determination of the effective dose of the applied RSK-I.
THP-1 cells were incubated for 24 h with PMA together with increasing doses of RSK-I. Subsequently, LAP*/LAP protein amounts were assessed (n=3).
