# Supplementary material for: C/EBPβ-LAP*/LAP Expression Is Mediated by RSK/eIF4B-Dependent Signalling and Boosted by Increased Protein Stability in Models of Monocytic Differentiation
Source: PLoS One. 2015 Dec 8;10(12):e0144338. doi: 10.1371/journal.pone.0144338 (PMC4672875; doi:10.1371/journal.pone.0144338)
Supplement: S2 Fig — THP-1 cells were incubated for 24 h with PMA together with increasing doses of PKR-I. Subsequently, LAP*/LAP protein amounts were assessed (n = 3). Additional lanes showing the effect of PKR-I on unstimulated cells have been removed (thin lines). (PDF) [file pone.0144338.s002.pdf]

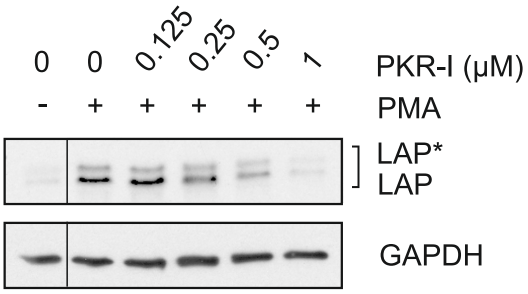

S2 Figure. Determination of the effective dose of the applied PKR-I.
THP-1 cells were incubated for 24 h with PMA together with increasing doses of PKR-I. Subsequently, LAP*/LAP protein amounts were assessed (n=3). Additional lanes showing the effect of PKR-I on unstimulated cells have been removed (thin lines).
