# Supplementary material for: C/EBPβ-LAP*/LAP Expression Is Mediated by RSK/eIF4B-Dependent Signalling and Boosted by Increased Protein Stability in Models of Monocytic Differentiation
Source: PLoS One. 2015 Dec 8;10(12):e0144338. doi: 10.1371/journal.pone.0144338 (PMC4672875; doi:10.1371/journal.pone.0144338)
Supplement: S3 Fig — THP-1 cells were treated with PMA up to 24 h and the trypsin-like activity of the proteasome was determined in whole cell extracts using protease-specific substrates (mean±SD; n = 3, duplicates). (PDF) [file pone.0144338.s003.pdf]

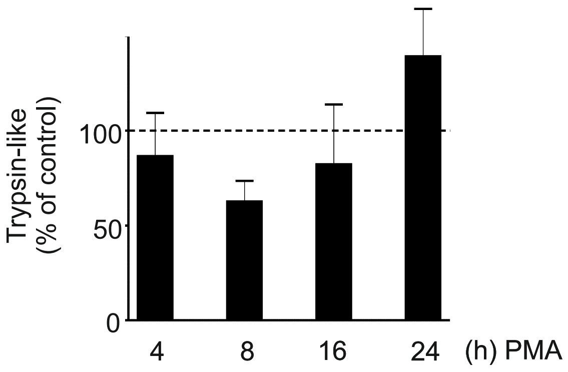

S3 Figure. Trypsin-like proteolytic activity in differentiating THP-1 cells.
THP-1 cells were treated with PMA up to 24 h and the trypsin-like activity of the proteasome was determined in whole cell extracts using protease-specific substrates (mean±SD; n=3, duplicates).
